# A Biodegradable Bioactive Glass-Based Hydration Sensor for Biomedical Applications

**DOI:** 10.3390/mi14010226

**Published:** 2023-01-15

**Authors:** Amina Gharbi, Ahmed Yahia Kallel, Olfa Kanoun, Wissem Cheikhrouhou-Koubaa, Christopher H. Contag, Iulian Antoniac, Nabil Derbel, Nureddin Ashammakhi

**Affiliations:** 1 CEM Lab, National Engineering School of Sfax, Sfax University, Sfax 3018, Tunisia; 2 Technopole de Sfax. BP 275, LT2S Lab, Centre de Recherche en Numérique de Sfax, Sfax 3000, Tunisia; 3 MST, Chair for Measurement and Sensor Technology, Technische Universittät Chemnitz, 09111 Chemnitz, Germany; 4 Institute for Quantitative Health Science and Engineering (IQ) and Department of Biomedical Engineering (BME), Michigan State University, East Lansing, MI 48824, USA; 5 SIM, Faculty of Material Science and Engineering, University Politehnica of Bucharest, 313 Bucharest, Romania

**Keywords:** bioactive glass, biodegradable, brain edema, capacitive sensor, hydration monitoring

## Abstract

Monitoring changes in edema-associated intracranial pressure that complicates trauma or surgery would lead to improved outcomes. Implantable pressure sensors have been explored, but these sensors require post-surgical removal, leading to the risk of injury to brain tissue. The use of biodegradable implantable sensors would help to eliminate this risk. Here, we demonstrate a bioactive glass (BaG)-based hydration sensor. Fluorine (CaF_2_) containing BaG (BaG-F) was produced by adding 5, 10 or 20 wt.% of CaF_2_ to a BaG matrix using a melting manufacturing technique. The structure, morphology and electrical properties of the resulting constructs were evaluated to understand the physical and electrical behaviors of this BaG-based sensor. Synthesis process for the production of the BaG-F-based sensor was validated by assessing the structural and electrical properties. The structure was observed to be amorphous and dense, the porosity decreased and grain size increased with increasing CaF_2_ content in the BaG matrix. We demonstrated that this BaG-F chemical composition is highly sensitive to hydration, and that the electrical sensitivity (resistive–capacitive) is induced by hydration and reversed by dehydration. These properties make BaG-F suitable for use as a humidity sensor to monitor brain edema and, consequently, provide an alert for increased intracranial pressure.

## 1. Introduction

Brain edema can develop following trauma and surgery, and can lead to increased intracranial pressure (ICP) and subsequent serious complications, including death [1]. Therefore, a number of approaches for continuous intracranial monitoring have been investigated [2]. The use of implantable sensors can be useful; however, they need to be removed after recovery, and this is associated with an increased risk of inflicting injury to the brain [3]. The development of biodegradable sensors for this purpose would offer a significant advantage, obviating the need for a second procedure for device removal [4]. Biodegradable sensors are largely polymer-based [5]; however, the hydrophilicity of most polymers leads to swelling and water absorption, which can cause premature failure of sensors due to early degradation of internal components. In addition, polymer degradation is associated with inflammatory responses [6,7,8], driving a need for alternative materials for intracranial sensing.

Biodegradable metals have been explored as the basis for intracranial sensors, but the constraints on their use include the accumulation of degradation by-products, which can have toxic effects [9]. Silicon (Si)-based materials can also be used in implantable sensors [10]; however, their degradation can produce reactive oxygen (O) species that cause cell death [11]. Due to its slow degradation and dielectric properties, silicon oxide (SiO_2_) has been used mainly as a sensor encapsulating and interlayer dielectric, which has been used in active neural interfaces [12]. In this context, most studies on Si-based implantable sensors have demonstrated their effectiveness in detecting parameters such as changes in pressure, temperature and pH [9]. In addition, Si-based biomaterials have the potential to contribute significantly to the development of new dielectric matrix concepts, mainly because the thermal, mechanical and optical properties of these amorphous structures are controllable [13]. However, bioceramics used in sensors generally have low reactivity, which is not recommended for use in monitoring brain edema that develops after surgery or trauma because these sensors can also lead to an increase in ICP [14].

To address this need, we sought to develop a hydration monitoring sensor based on biodegradable Si-based bioactive glass (BaG), which has favorable dissociation kinetics and reactivity for this clinical application, and when controlled associations with chemical elements are used, the physiological, mechanical and electrical effects can be modulated. However, in this context, the electrical sensitivity of BaGs is high, i.e., the capacitive character and the speed of electrical transfer mechanisms into the material matrix can easily be modified, and even completely altered. This has been proven through impedance measurements [15], where magnesium–zinc–calcium (MgZnCa) alloy hybrid glass was used as a material for the control of hydrogen release from biodegradable implants [16]. It was demonstrated that the absence of dislocation-based plastic-deformation resulted in favorable strength and elasticity compared with those of a competing crystalline Mg derivative [17]. Recently, a humidity sensor with high sensitivity based on cellulose nanofiber was developed but this device is not biodegradable [18].

Electrochemical impedance measurements of these types of materials were seen to provide a quick, direct and sensitive approach for the detection of DNA sequences using a silicon oxide (SiO_2_) transducer with peptide nucleic acid (PNA) as the probe layer [19]. Other structural studies of the current flow mechanism within the vitreous borosilicate matrix prove that the electrical conductivity, measured by impedance spectroscopy, increases with the amount of B_2_O_3_ added [20]. The activation energy of fluoride (F^-^), fluorophosphate and phosphate glass are similar when derived by conductivity or electric relaxation, presenting some deviation at the fluorophosphate type of glass. In addition, the conduction phenomenon based on the ionic-polaron hopping with the hopping rate being thermally activated was shown for all of these types of glass, whereas the impedance plots and activation energy evolutions show that the electrical characteristics follow a non-Debye behavior [21].

To extend these observations and develop a biodegradable BaG sensor, we intercalated CaF_2_ into SiO_2_-Na_2_O-CaO-P_2_O_5_. CaF_2_ is an insulator that may be integrated into microelectronics; moreover, its presence in the glass matrix may improve the rigidity of medical devices [22]. The *in vitro* and *in vivo* biocompatibility testing of another type of fluorine (CaF_2_) containing BaG (BaG-F) demonstrated an absence of inflammatory and toxic processes [23]. The chemical composition of our biomaterial may be controlled, enabling customization of their degradation. CaF_2_ intercalation can, therefore, be useful for developing implants with slower biodegradation [24,25]. In the current study, a novel BaG-based sensor was developed. To demonstrate how the chemical content of the glass materials affects density and electrical capacity, we evaluated several materials for biocompatibility properties. The electrical sensitivity of glass elements was found to be high and malleable, i.e., the capacitive character and the speed of the electrical transmission processes in the matrix of the material can easily be modified, and even completely eliminated [26]. Although this amorphous material possesses a lower ductility, it shows an appropriate Young’s modulus. The novelty of the material used for developing these sensors is based on the controllability of its composition—fluorine content in particular. Furthermore, in the fabrication procedure, we did not dope our materials with fluorine, but we reached the stage of substitution at the network modifiers level while keeping the vitreous structure and bioresorbability limits. Regarding its electrical distinction, the results obtained after the electrical impedance measurement in ambient air, BaG-F showed that the measured impedance could be modeled as a capacitor. It has a high sensitivity to changes in hydration (humidity), and therefore may be an exceptional candidate for monitoring brain edema and help to intervene before a serious increase in ICP develops and thus prevent death.

## 2. Materials and Methods

### 2.1. Material Fabrication

To evaluate the effect of CaF_2_ addition to BaG networks and to validate the fabrication protocol, a reference composition of BaG was employed [27]. BaG-F samples with varying F content were synthesized (Table 1).

**Figure 1:**
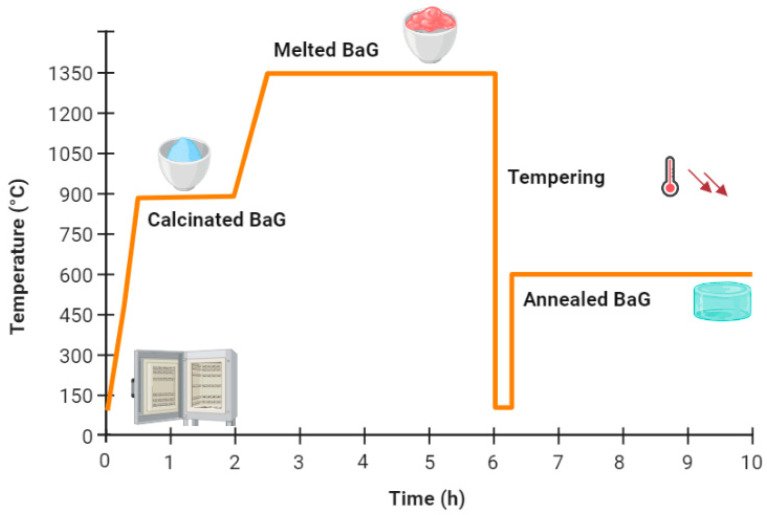
Thermal cycles used for bioactive glass (BaG)-based sensor. Some icons were taken from Biorender.com.

Appropriate contents of sodium metasilicate pentahydrate (Na_2_SiO_3_-5H_2_O, Aldrich, Darmstadt, Germany), CaF_2_ (Merck, Darmstadt, Germany), calcium metasilicate (CaSiO_3_, Merck, Darmstadt, Germany), sodium metaphosphate (Na_3_P_3_O_9_, Aldrich, Darmstadt, Germany) and calcium carbonate (CaCO_3_, Merck) were thoroughly mixed, and then subjected to a suitable thermal cycle (Figure 1). The molten vitreous material was then tempered in a preheated mold and annealed for 4 h at a temperature near to its glass transition temperature (Tg).

### 2.2. Structural and Morphological Analysis

For the identification of amorphous and crystallized phases in the materials studied, an AXS D8 ADVANCE diffractometer (BRUKER, Billerica, MA, USA) was used (voltage U = 30 kV and current I = 20 mA). The X-ray diffraction (XRD) patterns were recorded between 5 and 80 (2θ), in 20 min, with Cu Kα radiation. Scanning electron microscopy (SEM) using a Jeol JFC 1100 (ZEISS, Stuttgart, Germany) was carried out for imaging synthesized glass samples by scanning their surfaces with a fine electron stream. The BaG to be analyzed was metalized by a gold–palladium deposition (sputtering, Jeol JFC 1100) before being introduced into the analysis chamber. This metallization avoids the accumulation of charges on the surface of the samples (insulators) and reduces the penetration depth of the beam, which improves the image quality. The Brunauer–Emmett–Teller (BET) method was performed using an electronic analyzer (Flowsorb II 2300, Coultronics France SA, France) to determine the texture and specific surfaces of BaG-Fx disks by nitrogen physisorption. Specimens (100–150 mg) were placed in a cell mounted on the degassing compartment where they were heated under vacuum at 300 °C until the pressure reached 0.66 10^3^ –1.33 10^3^ Pa., then placed in the analysis compartment with successive adsorption and desorption of nitrogen as a function of the relative pressure P/P0.

### 2.3. Electrical Measurements

**Figure 2:**
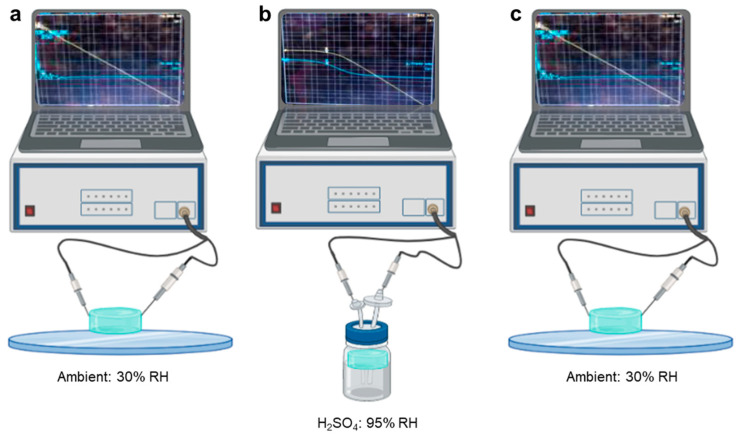
Measurement setup for fluorine bioactive glass (BaG-F)-based sensors showing the conductivity changes induced by the humidity created by potassium sulfate solution (H_2_SO_4_). (**a**) Capacitive character in ambient. (**b**) Resistive capacitive in high humidity. (**c**) Reversal of capacitive character in ambient environment. The icons were taken from Biorender.com. Curves were derived from our study in the figure.

The dielectric measurement was realized through a laboratory measurement system based on an impedance analyzer and copper electrode. Electrical measurements on BaG-F were performed using copper electrodes that were connected to the impedance analyzer (Agilent 4294A, Zeiss, Germany). Each surface of the BaG disc was attached to an electrode (Figure 2). BaG-F impedance was measured in the frequency range of 40 Hz to 30 MHz. The corresponding current was increasing with the frequency starting at current intensity in the pA range to 4 mA to better characterize the humidity impact on the electric behavior of BaG. To characterize the time dependence of the impedance (Z), BaG-Fx were kept under an atmospheric pressure of 1 atm at an ambient temperature and humidity of 25 °C and 30% RH, respectively (Figure 2a). The RH environment was formed using a saturated salt solution of potassium sulfate (K_2_SO_4_) 95% RH (Figure 2b). The measurements were carried out under ambient conditions for three minutes. 

The output can be a measure of the resistance (R) of the sensor by applying a direct current (DC), voltage (V) and measuring the DC current (I) through the sensor.

## 3. Results and Discussion

The manufacturing of BaG-F-based sensors was performed by varying the weight content of CaF_2_ in BaG to maximize the incorporation of F^-^ without destabilizing its network, and respecting the weight contents limits of each oxide to ensure the bioactivity and biodegradability of all BaG-F_x_ [28]. 

### 3.1. Physical Characterization of Fluorine Bioactive Glass (BaG-F) Based Sensors

#### 3.1.1. Structural Analysis

**Figure 3:**
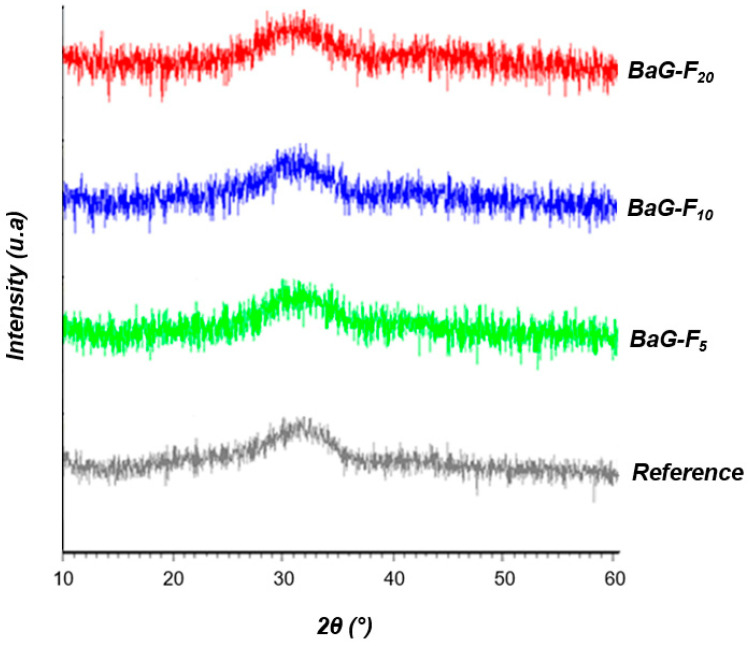
X-ray diffraction (XRD) patterns of fluorine bioactive glass (BaG-F_x_).

The XRD demonstrated phase stabilization of the fabricated BaG (Figure 3).

**Figure 4:**
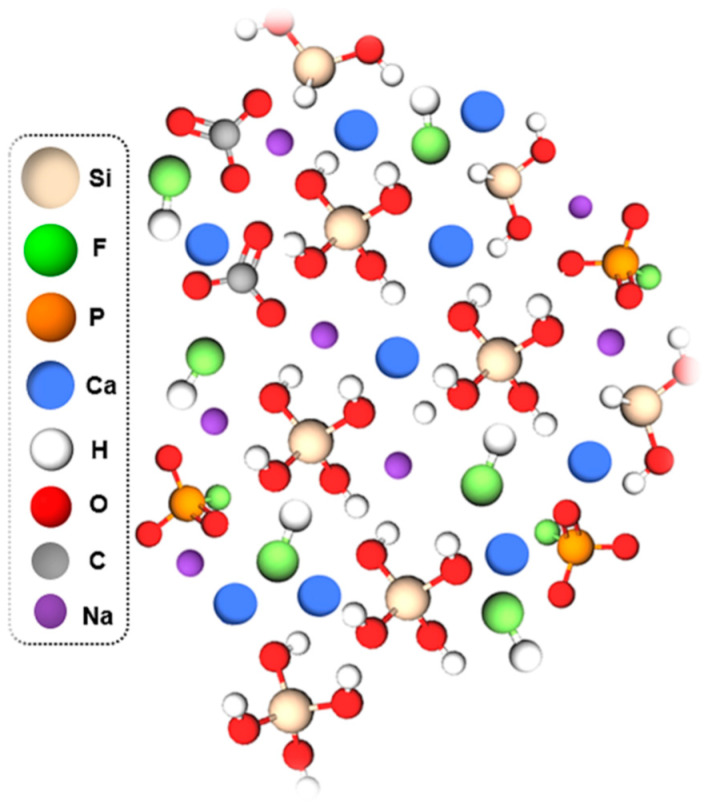
Investigated three-dimensional (3D) structure of fluorine bioactive glass (BaG-F_x_).

This type of halo (24°< 2θ <38°) corresponds to the diffusion phenomenon in amorphous materials [20]. Identification of crystalline phases by XRD was impossible for this type of material because of the periodicity absence of atomic arrangement within the vitreous network. No periodicity or stacking of planes at long distances, characteristic of pure glass. Therefore, the vitreous and stable structure of all BaG-F_x_ was proven, even after intercalation of CaF_2_ at a content of 20 wt.%. This promotes the phenomenon of electrical conductivity and fluid current flow through the free electrons in a purely disordered matrix that does not have boundaries of crystal reticular planes. Investigation of different molecular configurations of a disordered glassy network of BaG-F_x_ was carried out (Figure 4). This illustration provides information on the types of bonds that may be present in BaG-F_x_ [23]. For example, the formation of Si-F bonds or non-bridging fluorine is present. Furthermore, the presence of phosphorus, calcium and sodium increases fluorine reactivity in the matrix. The high concentration of non-bridging O resulting from Si-O bonding is evident. This means that if calcium fluoride is substituted for network modifiers such as CaO or Na_2_O, it results in the crosslinking of the network and an increase in network connectivity, thus promoting electrical transfer within the system.

The electronegativity and electronic configuration of elements that form and modify the network of the BaG (Table 2) may explain the shape of these molecular groups. The monatomic anion, F^-^, with its important electronegativity, will establish changes in the electrical conductivity between the chemical bonds of BaG-F_x_.

Understanding the atomic properties of each constituent element of this matrix type is important [29], to better explain the chemical bonding alterations that can affect glass morphology and its electrical behavior. Table 2 illustrates the atomic properties of each chemical element constituting the BaG-F network.

#### 3.1.2. Morphological Analysis

**Figure 5:**
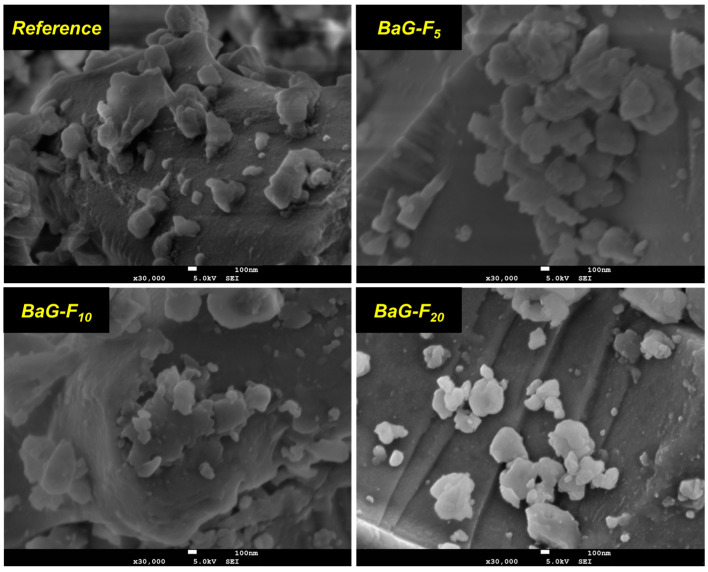
Scanning electron microscopy (SEM) images of fluorine bioactive glass (BaG-F_x_).

The micrographic SEM of BaG-F_x_ provided further insight into the direct effect of the insertion of F^-^ within the silicate–bioactive glass network being investigated. The SEM images (Figure 5) were in agreement with the previous XRD patterns. The morphology of BaG-Fx showed the presence of agglomerated particles, which indicate an amorphous and dense aspects with an absolute absence of pores. The surfaces of the BaG-Fx powders were formed by particles in the form of the monolithic islands’ propriety of an amorphous and dense structure. The added amount of CaF_2_ promoted the densification of BaG-Fx.

#### 3.1.3. Texture and Specific Surfaces Measurements

The specific surface areas (S_BET_), specific pore volumes (V_BET_), and pore diameters (D_pore_) of powders were measured by the multipoint BET method. Each BaG-F was degassed at 250 °C before measuring its specific surface area by BET. The specific surface values were obtained from the measurement of the nitrogen uptake isotherm (Table 3).

A decrease in specific surface areas and pore volumes with increasing CaF_2_ content was observed (Table 3). It should be noted that the values are higher than that of the pure (reference) glass. This proves that the incorporation of CaF_2_ is a critical factor in the morphological appearance of BaG-F_x_. This leads to concluding that the CaF_2_ addition to the BaG network results in micrometric powders with lower pore volumes than those obtained for reference. These results are in agreement with the observations made from SEM micrographs of BaG-F_x_.

**Figure 6:**
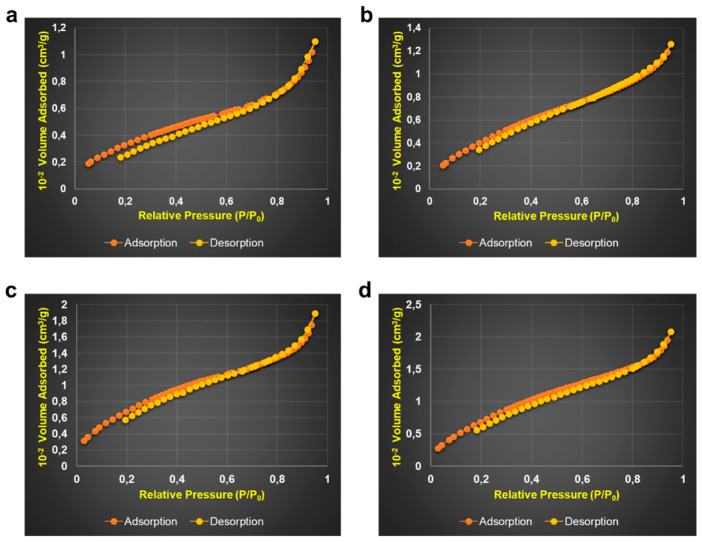
Adsorption–desorption isotherms of fluorine bioactive glass (BaG-F_x_). (**a**) Reference. (**b**) BaG-F_5_. (**c**) BaG-F_10_. (**d**) BaG-F_20_.

It was found that porosity decreases and grain size increases with increased CaF_2_ content in BaG matrix. As a result, the pore size observed on the surface of BaG-F_x_ samples is much smaller compared to the pore size in reference. Based on recent work on another type of BaG in a quaternary system [30], it is possible to precisely explain the results observed in the current study. Indeed, the morphology, shape and size distribution of microparticles were very sensitive to an added amount of CaF_2_. Recent studies have explained the effect of introducing CaF_2_ into glass microstructures [31,32,33]. On the other hand, the choice of initial mixture oxides, the good operating conditions of calcination and annealing, and the precious handling of BaG tempering allowed for a stable amorphous structure. CaF_2_ incorporation into a BaG network leads to its densification. Consequently, a decrease in porosity was observed with CaF_2_ [34]. These observations were confirmed by our results (Figure 5). It is well documented that the specific surface area has an important effect on the densification and bioactivity of material; as particle size increases, specific surface area decreases [35]. The values achieved were similar to those obtained in a previous study [36]. These measurements confirm analytical results obtained by SEM; porosity led to an increase in the specific surface area [37]. In agreement with our results, the presence of CaO favors the formation of pores [38]. Absorbing isotherms of fabricated BaG-F_x_ that are of reversible type II, characteristic of non-porous and microporous (< 2nm) adsorbent materials demonstrate the impact of adding CaF_2_ to BaG on the microstructure of the resulting constructs (Figure 6a–d). There was, a shift in the desorption curves occurring with a relative increase in the pressure.

This observation is in accordance with the results obtained, showing that pore diameter and specific surface decreases with CaF_2_. According to these isotherms, we can classify the porosity of studied BaG-F_x_ as type II [39].

### 3.2. Electrical Behavior of Fluorine Bioactive Glass (BaG-F) Based Sensors

In this investigation, it was assumed that the measurement area is the same for all BaG-F_x_. The choice of two limiting values of RH (minimum and maximum) to test the humidity sensitivity for our BaG was intentional. If a high sensitivity in this wide range is obtained, electrical functionality for intermediate values can be proved. The plan is to continue with more values in the next study to identify the effect of humidity as a function of conductivity and pressure based on digital simulation models. The output can be a measure of the resistance of the sensor by applying DC voltage and measuring the DC current through the sensor. In this case, it is more convenient to measure the conductance as a function of the resistance the: Y (Ω^−1^) = 1/R.

For ambient air, R is infinite. This is why we propose to measure Y with a DC current, as shown in Equation (1):

(1) $$Y=\frac{1}{R}=\frac{I}{V}$$

In this instance, for small values of the conductance (Y), small values of the humidity are observed, while for the high values of the conductance, humidity takes high values.

**Figure 7:**
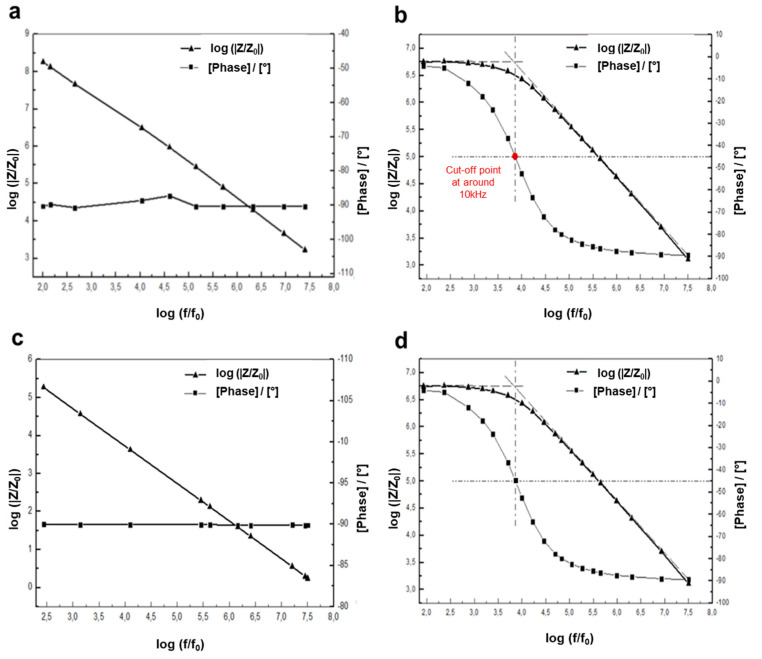
Reversible resistive–capacitive behavior for a bioactive 10% fluorine glass-based sensor (BaG-F_10_) during 30 min of measurement. (**a**) Capacitive character in ambient air. (**b**) Resistive–capacitive character in humidity (95% RH). (**c**) After the BaG-F_10_ is moved to the ambient environment, the capacitive character returns within 5 s. (**d**) Its resistive–capacitance characteristics can be reversed by re-contacting BaG-F_10_ with humidity. (f_0_ = 1 Hz, |Z_0_| = 1 Ohm).

The material (BaGs) to be examined is connected to a resistance, inductance and capacitor (RLC) meter or impedance analyzer. This technique is a simple, fast and non-destructive investigation that provides data on the dielectric properties of new materials subjected to an electric flow. All BaG-F_x_ samples were analyzed over a wide frequency range from 50 Hz to 30 MHz, generated at a constantly applied voltage of 500 mV (peak-to-peak). The frequency range included lower frequencies in order to properly select the sensitivity of this new material. For all BaG-Fx samples, a capacitive character in the ambient air was detected with a response time of about 8 s for either 3 or 30 min of measurement time. The measured impedance could then be modeled as a capacitor (*C*) (Figure 7a). The impedance (Z) can be calculated from the expression for the modulus in Equation (2):

(2) $$\left|Z\right|=\frac{1}{C\omega }$$

In this process, free movement of atomic electrons on the BaG-F_x_ surface ensures the flowing ionic charge transfer, especially since our BaG network was based on a semi-conducting chemical element which is Si. Moreover, F^-^ is at the top right of the chemical periodic table being the most electronegative atom (Table 2). It has a great tendency to extract itself from the valence layer by bonding with another free electron reaching the surface. Consequently, the presence of a reactive element (F^-^) in the glass insures a perfect electrical charge succession between network fragments of BaG-F_x_. In addition, energy provided by electric flow is sufficient for the decomposition of CaF_2_ due to the loss of one F^-^ atom from each F^-^ containing molecule. This desorption at the Si/CaF_2_ interface is manifested by breaking of Si-F bonds seen on IR examination. This results in the creation of Si-Ca which promotes electroconductivity elicited by calcium cation (Ca^2+^).

On the other side, measurements carried out in the ambient air for BaG-F_x_ showed that in capacitive developments, the phase is constant and relatively fixed at −90°. This outcome is a logical result, as such a negative phase is usually associated with capacitive performance. Besides, it can be stated that positive phase shifts can be obtained by inductive components [40]. Generally, bioactive electronic systems have to meet high sensitivity to physiological changes (changes in pH, water presence and temperature). In addition, special methods are needed to manufacture delicate medical devices to avoid their hydrolytic disintegration that may take place during the fabrication process [41]. In this context, the focus was on studying the effect of humidity on the electrical characteristics of BaG-F_x_. 

With the same electrical parameters as measurements made in ambient air, rapid change seen in the electrical behavior of BaG-F_x_ at 95% RH is interesting, and warrants further exploration of their response to water content. Precious electrical behavior has been observed for this type of biomaterial, for example BaG-F_10_ (with an average medium CaF_2_ added amount, Figure 7b).

In fact, BaG-F_10_ showed two behaviors, before the cutoff point, the resistive character predominates. Whereas for frequencies above this critical frequency point, which corresponds to approximately the −45° phase value, material capacitance power was high, and the capacitive aspect persisted. It has been proven that the apparent resistance and capacitance of materials strongly influence the magnitude and phase of low frequencies [42]. In the frequency range before cutoff point, the impedance (Z) of BaG-F_x_ can be equated to a resistance, while capacitive effects can be neglected before the cutoff point. Biomaterials have a resistive character, and the phase is zero. Therefore, Z is equal to R. For the frequency range above 10 kHz, Z can be represented as an equivalent circuit representing the capacitance (C) in parallel with the resistance (R). R and C are generated by a humidity-measuring device. In this range, the impedance (Z) can be determined using Equation (3):

(3) $$\left|Z\right|=\frac{R}{\sqrt{1+{\left(Rc\omega \right)}^{2}}}$$

This experiment proves that whatever the measurement duration of 3 or 30 min, BaG-F_10_ has a resistive and then a capacitive character (depending on the frequency range), and if it is removed from the humidity, i.e., water molecules (H_2_O) presence, it will quickly get free of adsorbed water and it will return to its initial capacitive character within 5 s (Figure 7c). This reversible behavior (Figure 7a–d) is due to the high reactivity of BaG-F_x_ versus humidity. 

To evaluate the effect of low (30%) and high (95%) levels of humidity on the conductance (Y) and DC current (I) through BaG-F, measurements at a DC voltage (V) of 5 V showed a large gap in conductance (0 to 0.17 10^−6^ Ω^−1^) and in current (0 to 0.85 µA) corresponding to low and high levels of humidity. respectively.

**Figure 8:**
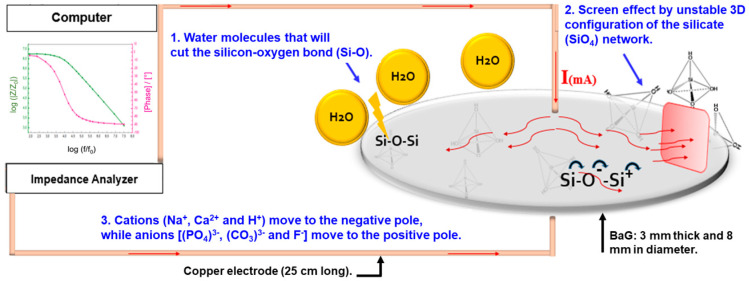
Illustration of the physicochemical mechanism behind the resistiveؘ–capacitive behavior of biomaterials. Humidity-induced distortion of the vitreous network favors an unstable resistivity because of the screen effect. (f_0_ = 1 Hz, |Z_0_| = 1 Ohm).

The same behavior was seen with the other BaG-F_x_ glass. This study adapts the hypothesis that surface contact of BaG-F_x_ has been affected by chemical bond type changes and molecular reorganizations. Through the physical adsorption mechanism, the charge exchange between H_2_O and BaG-F_x_ is well performed. From the first point of view, chemical contact into the H_2_O/BaG-F_x_ surface is known as a hydrolysis reaction [43]. SiO_2_ is the main BaG-forming compound configured as a vitreous network with tetrahedral units, and the four O atoms in silicate tetrahedron (SiO^-^_4_) are shared [20]. By hydrolysis, Si-O bonds are typically broken (step 1 in Figure 8). Depolymerization results in the formation of orthosilicates or the silanol group Si(OH)_4_ [44]. The aggregation of these groups forms a three-dimensional (3D) layer that acts as an insulating screen for the current (step 2 in Figure 8), giving the biomaterial a resistive character in the frequency range below the 10 kHz cutoff point (Figure 7b). Moreover, the Si(OH)_4_ hydroxide network acts as an insulating block due to its steric hindrance, which limits the electrical conductivity in the low-frequency range. However, these silicon hydroxides are not stable until polymerization by the liberation of H_2_O ions and then dehydration to return to the mis-ionic Si-O-Si bond takes place (step 3 in Figure 8). Si-O-Si is the active monomer that assures a smooth electrical transfer in the high-frequency range [28]. In addition, the high electronegativity of O and Si (Table 2) favors the current flow through this Si-O-Si siloxane bridge (Figure 8). 

From the second point of view, F^-^ plays an important role in these structural changes upon BaG contact with water. Recent research proves a reversible decrease in voltage as a function of the content of CaF_2_ added to the silicate network (SiO_2_) through the electrical measurements on the CaF_2_/SiO_2_ interface of ionic/covalent bond, respectively [45]. This process results from the substitution of CaF_2_ by CaO at the interface. CaF_2_, being very reactive during the electrical bombardment, provides the dioxygen (O_2_) for the exchange to take place. This allows the highlighting of hysteresis phenomena and negative differential resistance behavior for fluorine–silicate material. These dynamic effects could be modeled using an equivalent electrical circuit made of discrete elements (resistors–capacitors, in accordance with our results). An attractive alternative is the newly developed biodegradable ceramic-based sensor, which is capable of monitoring changes in ICP [46]. The major challenge with these implantable sensors is ensuring their stability throughout the clinically relevant monitoring period. It is challenging to have consistent electrical behavior from biodegradable sensors while they are in contact with body fluids, as early degradation will interfere with their behavior and function. Therefore, testing of these sensors over several weeks is required to define their suitability for different clinical applications and related operation times. Since the lifetime of the sensor relies on the chemical stability of the sensor material, which is determined by its degradation rate, the choice of the material and sensor design need to be carefully considered. To prolong the lifetime of sensing devices, thicker layers of the material need to be used. 

For this purpose, a new BaG with a controllable chemical composition that undergoes complete degradation *in vitro* in three months, was therefore, developed in the current study. Earlier, SiO_2_-based materials were used for making biodegradable sensors, which were functional for 22 days [47,48]. In summary, biodegradable The accuracy of ICP monitoring sensors in detecting clinically relevant ranges was demonstrated using *in vitro* studies. *In vivo* experiments, though limited, demonstrated the performance of these sensors for periods of 25 days in rats’ intracranial space. Long-term animal studies are required before clinical applications can be carried out. So far, no clinical translation of ceramic-based biodegradable has been reported yet.

In the present investigation, measured humidity (hydration) was investigated. This will potentially be useful to evaluate situations where edema may develop, such as may occur in the brain following trauma or surgery. Consequent to edema development, ICP increases, which can be associated with serious risks. Therefore, the capability to monitor brain edema using a biodegradable BaG-based sensor will be very useful. However, this needs to be demonstrated using specific models and animal experiments, which are planned to follow. 

## 4. Conclusions

It is possible to fabricate fluorine-containing bioactive glass, which is characterized by stability, high density and electrical conductivity. It is possible to have hydration-induced electrical activity behavior of the developed BaG-F (becoming resistive and then conductive), which can be reversed upon dehydration. Because of their structural, chemical and electrical properties, Bag-F implantable devices can serve as sensors. This is the first study to demonstrate the potential of BaG-F to serve as a sensor for the monitoring of hydration and humidity. The results of this study, therefore, may provide a potential candidate for monitoring of brain edema and addressing an important clinical problem. Further simulation studies are in progress to optimize this sensor further (matching conductivity and humidity). 

## Figures and Tables

**Table 1:** Chemical compositions of fluorine bioactive glass (BaG-F_x_: 0 < x < 20 wt.%).

BaG	SiO_2_	CaO	Na_2_O	P_2_O_5_	CaF_2_
Reference	46	24	24	6	0
BaG-F_5_	46	22	23	4	5
BaG-F_10_	45	21	22	2	10
BaG-F_20_	41	18	20	1	20

**Table 2:** Physical and atomic properties of the components of the fluorine bioactive glass (BaG-F_x_).

Chemical Element	Valence Layer	Electronegativity (Pauling Scale)	Oxydation State	Electrical Conductivity (s·m^−1^)
^14^Si	3s^2^ 3p^2^	1.9	+1, +2, +3, +4	2.52 × 10^−4^
^9^F	2s^2^ 2p^5^	3.98	−1	-
^8^O	3s^2^ 3p^4^	3.44	−2, −1	-
^20^Ca	4s^2^	1	+2	29.8 × 10^6^
^11^Na	3s^1^	0.93	+1	21 × 10^6^
^15^P	3s^2^ 3p^3^	2.19	±3, 5, 4	1.0 × 10^−9^

**Table 3:** Brunauer–Emmett–Teller (BET) data for fluorine bioactive glass BaG-F_x_.

	Specific Surface (m^2^·g^−1^)	Pore Diameter (Å)	Specific Pore Volume 10^−3^ (cm^3^·g^−1^)
Reference	1.205 ± 0.03	52.11 ± 0.3	1.630 ± 0.04
BaG-F_5_	1.004 ± 0.03	49.37 ± 0.3	1.185 ± 0.04
BaG-F_10_	0.704 ± 0.03	46.2 ± 0.3	0.834 ± 0.04
BaG-F_20_	0.098 ± 0.03	40.05 ± 0.3	0.073 ± 0.04
